# Cancer-Related Anemia Is a Risk Factor for Medium-Term Postoperative Cognitive Dysfunction in Laparoscopic Surgery Patients: An Observational Prospective Study

**DOI:** 10.1155/2020/4847520

**Published:** 2020-02-06

**Authors:** Huimei Huang, Fei Lin, Liming Cen, Ren Jing, Linghui Pan

**Affiliations:** ^1^Department of Anesthesiology, Guangxi Medical University Cancer Hospital, Nanning 530021, Guangxi Zhuang Autonomous Region, China; ^2^Perioperative Medicine Research Center, Guangxi Medical University Cancer Hospital, Nanning 530021, Guangxi Zhuang Autonomous Region, China

## Abstract

Anemia in the elderly may impair cognitive function. Our primary objective was to determine whether cancer-related anemia was associated with postoperative cognitive dysfunction (POCD) in nonelderly patients. We conducted an observational prospective study of 177 patients scheduled for laparoscopic surgery. Patients aged 18-64 were divided into two groups according to whether they were anemic due to cancer or not. The cognitive function was assessed by the Mini-Mental State Examination (MMSE) 1 day before and 1 week after operation. The cognitive function of the patients was evaluated by using the Telephone Interview for Cognitive Status-Modified (TICS-M) 3 months after operation. The quality of life of patients was evaluated after operation. The hemoglobin level and other clinical data were recorded before operation. Of the 170 patients, 100 without anemia and 70 anemia patients had been evaluated 1 week after operation. POCD was detected in 43 cases (25.3% of 170 cases) at 1 week and 30 cases (19% of 158 cases) at 3 months postoperatively. Anemia was an independent risk factor for 3-month POCD occurrence (*P* = 0.034). The education level of the patients who had POCD at 1 week and 3 months after operation was lower (*P* < 0.001, *P* = 0.011, respectively). Age was independently associated with the incidence of POCD at 3 months (*P* = 0.011). In general, these findings suggested that anemia may increase the incidence of medium-term POCD in cancer patients undergoing laparoscopic surgery.

## 1. Introduction

Postoperative cognitive dysfunction (POCD) is a common postoperative complication, affecting many cognitive domains, including attention, memory, executive function, and information processing speed. These impairments seriously affect the quality of life and increase the risk of disability and mortality [1–3]. Although age is considered to be an independent risk factor for POCD, it can occur at any age [4]. A previous trial recorded that POCD was present in 36.6% of the young (18-39 years old), 30.4% of the middle-aged (40-59 years old), and 41.4% of old patients (60 years old or above) at the time of discharge [3]. Although POCD is generally considered to be of clinical importance, its specific pathogenesis is still unclear, and there is not enough effective treatment [5]. Therefore, the most effective and economic treatment strategy is to study the risk factors for POCD and early clinical intervention.

Cancer-related anemia (CRA) is the most common complication of malignant tumors. Cancer causes malnutrition of body absorption, and long-term radiotherapy and chemotherapy can also lead to CRA. Recent studies have shown that age [6], diabetes [7], preexisting cognitive impairment [8], and educational level [9] are risk factors for POCD. However, the effect of anemia on POCD remains controversial. Weiskopf et al. [10] found that even in healthy nonsurgical subjects, acute anemia can cause cognitive impairment. A systematic review showed a significant positive correlation between anemia and overall cognitive decline, decreased executive function, and the incidence of dementia [11]. Joosten and colleagues report that anemia is an independent risk factor for delirium and dementia in hospitalized elderly patients [12]. However, a recent report shows that no correlation between anemia and cognitive outcomes had been found among older people in an acute surgical setting [13]. These findings indicated that anemia might have a negative impact on POCD in the elderly patients, but the effect of anemia on POCD in young patients remains unclear. What is more, all the above studies are retrospective; prospective studies are needed to further illuminate the relationship between anemia and POCD.

Thus, we conducted a prospective study to evaluate the correlation between the anemia and POCD in young patients undergoing laparoscopic surgery.

## 2. Materials and Methods

### 2.1. Participants

This prospective observational study was conducted in Guangxi Medical University Cancer Hospital for 12 months in 2018. The research ethics license (no. KY2018001) was provided by the ethics committee of Guangxi Medical University Cancer Hospital on February 5, 2018. This study was registered in China Clinical Trial Registration Center (ChiCTR1800015123), and all patients obtained informed consent. A total of 177 patients with gastrointestinal or gynecologic cancer were scheduled to undergo laparoscopic surgery. All the participants were divided into a normal group and an anemia group (Hb < 120 g/L in male, Hb < 110 g/L in female) according to hemoglobin before operation.

Approximately 1 day before operation, patients were assessed and screened. Cancer-related anemia was defined as the presence of anemia in cancer complications. Patients, aged between 18 and 64, with American Society of Anesthesiologists (ASA) physical status I to II, hemoglobin ≥ 60 g/L, and elective or limited tumor laparoscopic surgery under general anesthesia were consecutively recruited for the study. Exclusion criteria included MMSE score < 24, history of depression, schizophrenia, epilepsy, Parkinson's disease, myasthenia gravis, severe Alzheimer's disease, any serious visual or auditory disorders, language understanding disorders, coma, end-stage diseases, emergency operation, and neurosurgery history.

### 2.2. Procedures and Anesthesia

Demographic information (age, gender, height, and weight), comorbidity rate, ASA physical condition, hemoglobin, and MMSE scores were recorded before operation. End expiratory carbon dioxide partial pressure, urine volume, blood loss, and blood transfusion volume were intraoperatively monitored. All patients received the same anesthesia, and no drug was given before operation. Anesthesia was induced by intravenous sufentanil or fentanyl, rocuronium, or cisatracurium. During surgery, propofol was used to maintain anesthesia to achieve a bispectral index of 40 to 60 points. Avoid inhaling the anesthetic during operation. Patients who received sufentanil were titrated according to clinical requirements. Discontinue the use of cisatracurium or rocuronium to maintain adequate muscle relaxation. All the patients received the same postoperative pain control protocol of patient-controlled analgesia (PCA, constant speed 2 mL/h, locking time 15 min) with sufentanil 0.03-0.05*μ*g/(kg·h) plus tropisetron 10 mg for 2 days.

### 2.3. Postoperative Assessment of Cognitive Function and Quality of Life

In order to evaluate cognitive function, MMSE was used before anesthesia (to obtain cognitive baseline) and 1 week after surgery or at discharge (short-term POCD), and TICS-M was used at 3 months (medium-term POCD). The tests were conducted in a peaceful environment at the same time of the day, as determined by the same researcher. POCD was diagnosed when the MMSE score was declined 1 SD or more at 1 week after operation compared with the preoperative MMSE score [13, 14] or TICS-M score < 33 at 3 months after operation.

The Chinese version of the EORTC core quality of life questionnaire (EORCT QLQ-C30) was used to evaluate the patients' postoperative quality of life. The EORTC QLQ-C30 had a total of 30 scoring entries, including 5 functional areas, 3 symptom areas, 1 overall health status/quality of life area, and 6 single field items.

### 2.4. Statistical Analysis

According to previous reports, the incidence of POCD varies from 30% to 60% after cardiac surgery [15–17]. We assumed that the incidence rate of POCD in young patients undergoing laparoscopic surgery is lower than that in patients undergoing cardiac surgery, and the incidence of anemia is higher than that in normal patients. Thus, we estimated that the incidence rates of POCD in normal and anemic patients are 20% and 40%, respectively. A sample size of 165 would allow us to detect differences with a power of 0.80 at a 0.05 significance level. Considering a 5% miss visiting rate, we intended to include a total of 174 patients in our study.

Numerical variables were presented as mean and standard deviation ($\bar{X}\pm S$), and the difference in mean values between the two groups was assessed by the *t*-tests. The *χ*^2^ test was performed for the comparison of the categorical data. Univariate logistic regression analysis was done to investigate the correlation between the covariance. Variables with *P* values < 0.2 were then added to a binary logistic regression model with a forward selection method to determine the probable risk factors associated with POCD. All statistical analyses were performed with Statistical Product and Service Solutions (SPSS, version 22.0). All *P* values are two-tailed, and *P* less than 0.05 was considered statistically significant.

## 3. Results

### 3.1. Basic Characteristics of Patients Included

**Figure 1:**
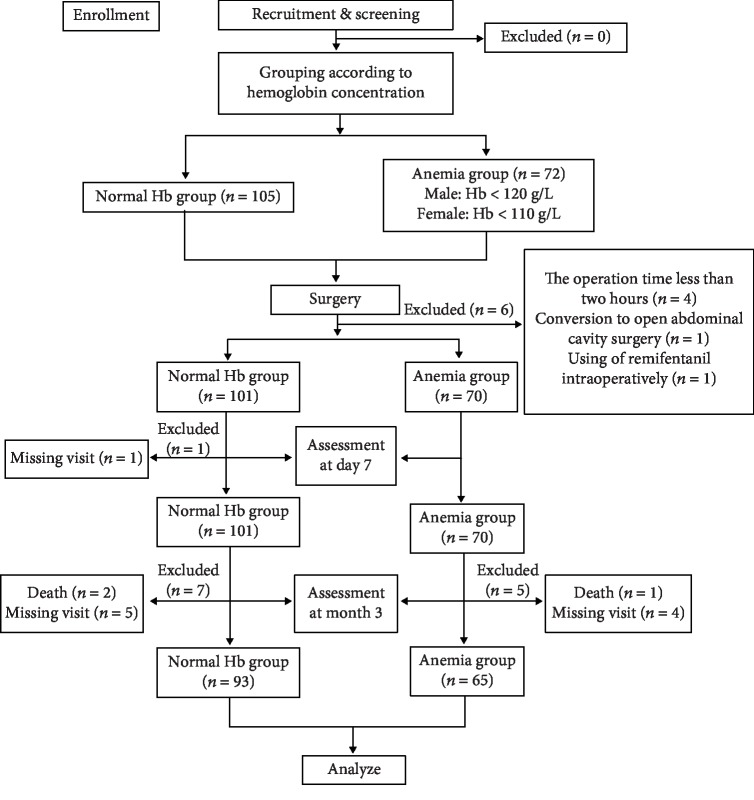
Patients included and excluded in our study.

There were 177 cancer patients undergoing laparoscopic surgery in this study, but 7 patients were excluded from the 7-day evaluation, including 4 patients whose operation time was less than 2 hours; 1 patient turned to open abdominal surgery; 1 patient used remifentanil during operation, and 1 patient was absent from the visit. As a result, 170 patients participated in the study. Among 170 patients, 70 (41%) were anemic, including 20 moderate anemia, 50 mild anemia, and 100 nonanemia. The study design is shown in Figure 1. The incidence rate of POCD at 1 week for all the patients was 25.3% (normal 21.0%, anemia 31.4%). The incidence rate of POCD at 3 months for all the patients was 19.0% (12.9% in normal patients and 27.7% in anemia patients). At 3 months after operation, 12 other cases were excluded: 3 died and 9 missed diagnosis.  Table 1 summarizes the basic characteristics of patients in the normal group and anemia group. There are no significant differences in age, gender, body mass index (BMI), education level, ASA grade, and previous medical history between the two groups (*P* > 0.05).

### 3.2. Evaluation of Postoperative Cognitive Function

We evaluated the MSEE scores of 70 anemic patients and 100 normal patients one day before and one week after surgery. There was no significant difference between the normal group and the anemia group in preoperative and postoperative scores (all *P* value > 0.05) (Table 2). The MSEE score of the two groups showed a downward trend. A total of 65 anemic patients and 93 normal people completed the TICS-M score evaluation 3 months after operation. The TICS-M score of the anemia group was lower than that of the normal group (*P* = 0.026) (Table 2).

### 3.3. Risk Factors for POCD

When studying the risk factors for POCD occurrence, univariate analyses were conducted to assess the relationship between clinical parameters and outcome of these patients. As shown in Table 3, education level (OR = 0.830, 95%CI = 0.748-0.921) and preoperative Hb (OR = 0.899, 95%CI = 0.821-0.984) were significantly associated with POCD occurrence in 1 week after operation (*P* < 0.001 and *P* = 0.022, respectively). Age (OR = 1.083, 95%CI = 1.024-1.146), education level (OR = 0.869, 95%CI = 0.776-0.973), preoperative K concentration (OR = 4.453, 95%CI = 1.331-14.897), preoperative Hb (OR = 0.903, 95%CI = 0.816-0.999), and anemia (OR = 0.387, 95%CI = 0.171-0.873) were significantly correlated with POCD occurrence in 3 months after operation (*P* = 0.006, 0.015, 0.015, 0.047, and 0.022, respectively).

### 3.4. Independent Risk Factors for POCD

To explore the independent risk factors for POCD, binary logistic regression was applied to these factors with *P* < 0.2 in the univariate analyses. Because of the possible collinear relationship between preoperative hemoglobin and anemia, we considered anemia rather than preoperative hemoglobin in binary logistic regression analyses. As shown in Table 4, education level (OR = 0.828, 95%CI = 0.746-0.919) was the independent factor of POCD occurrence in 1 week after operation (*P* < 0.001). Age (OR = 1.082, 95%CI = 1.019-1.150), education level (OR = 0.885, 95%CI = 0.758-0.964), and anemia (OR = 0.393, 95%CI = 0.165-0.932) were the independent factors for POCD occurrence in 3 months after operation (*P* = 0.011, 0.011, and 0.034, respectively).

### 3.5. Influence of Anemia on Postoperative Quality of Life

A total of 136 patients completed the EORCT QLQ-C30 assessment at 3 months after operation, including 57 anemia patients and 79 normal patients. In symptom areas, the score of fatigue in the anemia group was lower than that in the normal group (*P* < 0.05). There were no significant differences between the two groups in other symptom areas and functional areas (*P* > 0.05) (Table 5).

## 4. Discussion

The potential risk factors for POCD have not been fully elucidated. Moreover, there were few articles in this field to demonstrate the relationship between preoperative anemia and postoperative cognitive dysfunction. The aim of this study was to assess the short- and medium-term postoperative cognitive function of adult patients who, as part of their general anesthesia for elective laparoscopic surgery, had low hemoglobin levels preoperatively. Our preliminary result showed that patients with anemia were susceptible to POCD at 3 months after surgery, but this was not significantly correlated with the incidence of POCD at 1 week postoperatively.

In the present study of 170 laparoscopic surgery patients, the incidence of POCD at 1 week and 3 months was 25.3% and 19.0%, which were lower than that of the previous report [3]. The possible reason for the result was that the more young patients in our study, all of whom had laparoscopic surgery, had less trauma than those who had laparotomy and cardiosurgery. The incidence of POCD in anemic patients at one week and three months was higher than that in nonanemic patients (31.4% vs. 21.0% and 27.7% vs. 12.9%, respectively), suggesting that the incidence of POCD in anemic patients may be higher than that in nonanemic patients. Preoperative anemia was known to be associated with 30-day postoperative mortality (odds ratio 1.42, 95% CI 1.31-1.54) and postoperative morbidity (adjusted odds ratio 1.35, 95% CI 1.30-1.40) in surgical patients [14]. The presence of anemia and its role in determining cognitive function in elderly patients have been investigated in many studies, and the results showed that there was a significant association between anemia and cognitive impairment [11, 12]. Our preliminary results demonstrate that anemia may also lead to cognitive impairment in young patients.

The diagnosis of POCD remains controversial and lacks a unified diagnostic standard. The MMSE is a well-accepted global cognitive function measurement method, with sensitivity and specificity values of 80% to 95% and 86% to 100%, respectively, despite its obvious limitations (ceiling effect, performance affected by age and education) [18, 19]. In our study, we assess the cognitive function by using the Chinese version of the MMSE, which is consistent with many other studies [20]. When personal screening is impractical or patients are unable to go to the hospital for cognitive function assessment, TICS-M can be used by phone [21]. It has become the most widely used telephone screening tool [22], with sensitivity and specificity values of 82.4% to 99% and 86% to 87%, respectively [23, 24]. However, MMSE and TICS-M alone may not be enough for POCD diagnosis, which is one of the limitations of this study. Many other means for diagnosis of POCD should be used in future research, such as Wechsler Intelligence Scale (WIS) [25], Minnesota Multiphasic Personality Inventory (MMPI) [26], neuroelectrophysiological examination [27], and biochemical marker (such as IL-6, S-100*β*, and NSE) examination [28].

When investigating the independent risk factors for POCD occurrence, binary logistic regression analysis was applied. In our study, education was an independent risk factor for POCD at 1 week and 3 months (*P* < 0.001 and *P* = 0.011, respectively), which is consistent with previous research [9]. Age is an independent risk factor for POCD occurrence at 3 months (*P* = 0.011), which is consistent with the previous study, indicating that age is the most important risk factor for POCD [29]. The possible reason that age has a medium-term effect but the short-term POCD incidence is not statistically significant is that we recruited patients aged 18 to 64 years, excluding older patients in our study. Anemia is not an independent factor of POCD for 1 week (*P* > 0.05), but there was statistical significance in the incidence of POCD at 3 months (*P* = 0.034), indicating that anemia had a medium-term effect on cognitive impairment.

The EORCT QLQ-C30 assessment was used to compare the quality of life between the two groups. The results showed that the fatigue score of the anemia group was significantly lower than that of normal group patients, which may be due to insufficient cases in our study. There was no statistically significant difference in most of symptom areas and functional areas between the two groups of patients, indicating that anemia has little effect on the overall quality of life of the postoperative tumor patients. However, several studies have shown that cancer-related anemia and fatigue are important factors affecting the quality of life of cancer patients [30, 31]. The effect of cancer-related anemia on postoperative quality of life needs more sample study.

As we all know, the pathogenesis of POCD is multifactorial, including inflammatory stress responses, pain, environmental factors, and trauma. Central nervous injury mediated by central inflammatory response has been considered one of the important mechanisms of POCD [32]. A study [33] shows anemia may impair cognitive performance by decreasing brain oxygen delivery, with brain metabolism alterations. The possible reasons may be that hypoxia impaired neuronal protein synthesis and synaptic plasticity [34], which are essential for learning. Exposure to hypoxia triggered a series of cell survival-centered events.

There were some limitations in our study. First, we cannot enroll sufficient anemia patients, because these patients lack the opportunity of laparoscopic surgery. Second, the relatively short follow-up time limits our observation of the long-term cognitive impairment caused by anemia, and a more statistical influence may be found after longer follow-up. Third, the diagnosis of POCD still remains controversial.

## 5. Conclusions

In summary, our study has confirmed that anemia is not a sufficient risk factor for increasing the incidence of cognitive dysfunction in one week after surgery, but the incidence of cognitive dysfunction will increase in three months after surgery. Further large-scale trials are needed to evaluate the effect of anemia on the postoperative cognitive dysfunction.

## Figures and Tables

**Table 1:** Baseline, preoperative, and demographic characteristics of the two group patients.

Patient characteristics	1-week follow-up			3-month follow-up		
	Anemia (*N* = 70)	Normal (*N* = 100)	*P* value	Anemia (*N* = 65)	Normal (*N* = 93)	*P* value
Age (years)	50.54 ± 8.54	49.88 ± 8.08	0.406	50.57 ± 8.68	49.41 ± 8.053	0.326
Gender (male/female)	21/49	24/76	0.383	20/45	21/72	0.248
BMI (kg/m^2^)	22.41 ± 3.24	22.65 ± 3.24	0.393	22.27 ± 3.20	22.60 ± 3.31	0.403
Education (year)	8.90 ± 4.14	9.12 ± 3.61	0.471	8.98 ± 4.20	8.90 ± 3.59	0.545
ASA classification (I/II)	30/40	44/56	0.882	28/37	40/53	0.993
Preoperative chemotherapy (yes/no)	22/48	19/81	0.070	21/44	18/75	0.063
History of surgery (yes/no)	18/50	20/74	0.458	16/47	19/68	0.611
History of hypertension (yes/no)	6/62	10/84	0.794	6/57	8/79	0.946
History of diabetes (yes/no)	2/66	4/90	0.662	2/61	4/83	0.661

BMI = body mass index.

**Table 2:** The MMSE score and TICS-M score between the two group patients ($\bar{X}\pm S$).

	Anemia group	Normal group	*P* value
Preoperative MSEE score	26.84 ± 1.65	26.85 ± 1.56	0.621
Postoperative MSEE score	25.80 ± 1.93	26.14 ± 2.03	0.632
Postoperative TICS-M score	34.71 ± 4.17	35.91 ± 3.55	0.026

MMSE = Mini-Mental State Examination; TICS-M = Telephone Interview for Cognitive Status-Modified.

**Table 3:** Univariate analysis results for POCD at 1 week and 3 months after operation.

	POCD at 1 week		POCD at 3 months	
	OR (95% CI)	*P* value	OR (95% CI)	*P* value
Age (years)	1.034 (0.990-1.081)	0.134	1.083 (1.024-1.146)	0.006
Gender (male vs. female)	1.064 (0.483-2.343)	0.878	1.505 (0.568-3.993)	0.411
BMI (kg/m^2^)	1.053 (0.946-1.173)	0.343	1.091 (0.964-1.234)	0.167
Education (years)	0.830 (0.748-0.921)	<0.001	0.869 (0.776-0.973)	0.015
ASA classification (years)	0.750 (0.375-1.502)	0.417	0.954 (0.428-2.130)	0.909
Preoperative chemotherapy (yes/no)	0.559 (0.260-1.204)	0.137	0.586 (0.247-1.391)	0.225
History of surgery (yes/no)	1.119 (0.488-2.568)	0.791	0.442 (0.143-1.366)	0.156
History of hypertension (yes/no)	0.680 (0.184-2.519)	0.564	0.643 (0.136-3.040)	0.577
History of diabetes (yes/no)	1.553 (0.274-8.813)	0.619	0.793 (0.089-7.053)	0.835
Fasting blood glucose (mmol/L)	1.182 (0.930-1.502)	0.172	1.038 (0.796-1.354)	0.785
Preoperative Na concentration (mmol/L)	0.889 (0.737-1.097)	0.294	0.847 (0.670-1.070)	0.164
Preoperative K concentration (mmol/L)	2.270 (0.820-6.282)	0.114	4.453 (1.331-14.897)	0.015
Preoperative Ca concentration (mmol/L)	0.077 (0.003-1.722)	0.106	0.034 (0.001-1.102)	0.057
Preoperative Hb (g/L)	0.899 (0.821-0.984)	0.022	0.903 (0.816-0.999)	0.047
Anemia (yes vs. no)	1.724 (0.859-3.463)	0.126	0.387 (0.171-0.873)	0.022
RBC input (cases)	1.500 (0.265-8.493)	0.647	2.214 (0.386-12.694)	0.372
Plasma input (mL)	1.000 (0.996-1.004)	0.833	1.002 (0.999-1.006)	0.240
Total infusion (mL)	1.000 (1.000-1.001)	0.268	1.000 (0.999-1.000)	0.562
Hemorrhage during operation (mL)	1.001 (0.999-1.002)	0.270	1.000 (0.999-1.002)	0.613
Intraoperative urine output (mL)	1.000 (0.998-1.002)	0.824	1.000 (0.998-1.002)	0.728
Surgical site (midsection/hypogastrium)	1.175 (0.406-3.401)	0.767	0.929 (0.287-3.009)	0.929
Hospital stays (day)	1.008 (0.959-1.059)	0.759	0.994 (0.937-1.053)	0.830
The anesthesia time (min)	1.000 (0.996-1.004)	0.995	1.000 (0.995-1.005)	0.901
The operation time (min)	1.000 (0.996-1.005)	0.871	1.000 (0.995-1.005)	0.954

POCD = postoperative cognitive dysfunction; BMI = body mass index; RBC = red blood cell; HR = hazard ratio; CI = confidence interval.

**Table 4:** Multivariate analysis results for POCD at 1 week and 3 months after operation.

	POCD at 1 week		POCD at 3 months	
	OR (95% CI)	*P* value	OR (95% CI)	*P* value
Age (years)	—	—	1.082 (1.019-1.150)	0.011
BMI (kg/m^2^)			—	—
Education (years)	0.828 (0.746-0.919)	<0.001	0.855 (0.758-0.964)	0.011
Preoperative chemotherapy (yes/no)	—	—		
History of surgery (yes/no)			—	—
Fasting blood glucose (mmol/L)	—	—		
Preoperative Na concentration (mmol/L)			—	—
Preoperative K concentration (mmol/L)	—	—	—	—
Preoperative Ca concentration (mmol/L)	—	—	—	—
Anemia (yes vs. no)	—	—	0.393 (0.165-0.932)	0.034

POCD = postoperative cognitive dysfunction; BMI = body mass index; HR = hazard ratio; CI = confidence interval.

**Table 5:** EORCT QLQ-C30 scores between anemia group and normal group patients at 3 months after operation.

	Anemia group (*n* = 57)	Normal group (*n* = 79)	*P* value
Functional areas			
Body functions	87.13 ± 16.37	87.51 ± 13.39	0.261
Role functions	78.65 ± 19.35	76.16 ± 17.23	0.795
Emotional functions	88.59 ± 12.26	87.97 ± 12.78	0.951
Cognitive functions	92.69 ± 11.36	91.98 ± 9.19	0.196
Social function	79.53 ± 15.43	82.27 ± 15.17	0.822
Overall health status	78.50 ± 19.34	77.21 ± 15.65	0.385
Symptom areas			
Tired	19.29 ± 18.24^a^	24.05 ± 12.50	0.012
Nausea and vomiting	6.14 ± 13.22	10.54 ± 16.48	0.267
Pain	9.94 ± 12.93	10.54 ± 13.65	0.539
Shortness of breath	4.67 ± 11.68	7.17 ± 28.07	0.681
Insomnia	17.54 ± 20.02	17.72 ± 22.54	0.779
Loss of appetite	15.20 ± 20.95	16.45 ± 20.58	0.824
Constipation	2.92 ± 9.51	7.59 ± 17.66	0.344
Diarrhea	4.67 ± 15.98	5.06 ± 12.04	0.278
Economic hardship	46.19 ± 23.36	39.66 ± 26.72	0.342

## Data Availability

The data used and/or analyzed during the current study are available from the corresponding author on reasonable request.
